# Clinical and Pathological Manifestations with Differential Diagnosis in Behçet's Disease

**DOI:** 10.1155/2012/690390

**Published:** 2011-11-28

**Authors:** Aysin Kokturk

**Affiliations:** Department of Dermatology, Faculty of Medicine, Mersin University, Zeytinlibahçe 33079, Mersin, Turkey

## Abstract

Behçet's disease is a multisystemic inflammatory disease of unknown etiology which usually occurs as a trait of symptoms: aphthous stomatitis, genital ulcerations, and ocular disease. At the beginning of the disease the diagnosis is uncertain because of various clinical manifestations and a long period up to the full clinical picture manifestation. Since neither the laboratory data nor the histopathological signs are truly pathognomonic in Behçet's disease, the differential diagnosis depends on a careful evaluation of the medical history and meticulous physical examination to detect concomitant systemic manifestations. Sometimes, some laboratory test may help establish the diagnosis. Subspecialty referral to ophthalmology, rheumatology, neurology, and gastroenterology should be considered when indicated.

## 1. Introduction

Behçet's disease (BD) is a multisystemic inflammatory process of unknown etiology, characterized by relapsing episodes of oral aphthous ulcers, genital ulcers, other skin lesions, and ocular lesions. The Turkish dermatologist Hulusi Behçet first described the disease in 1937, as the triad of recurrent oral aphthous ulcers, genital ulcers, and uveitis [1]. It can affect nearly every system and organ including ocular, cardiovascular, gastrointestinal, renal, pulmonary, urologic, and central nervous systems and the joints [1–4]. 

It affects people mainly between the ages of 20 to 40. Both genders are usually equally affected [2, 3]. However different male-to-female ratio was recorded in some countries: there is a male predominance in Middle Eastern countries, such as Iraq, Jordan, Saudi Arabia, and Lebanon, while a female predominance is seen in the USA and Britain [4–8].

Because there are no specific diagnostic laboratory tests or histopathologic findings, the diagnosis of the disease relies on clinical criteria and often takes several years to establish a definitive diagnosis after the appearance of the initial manifestations. Manifestations of BD are not consistent among patients. Clinical phenotypes are very heterogeneous and evolution of the disease vary due to ethnic, geographical, and individual differences. 

Moreover, the initial manifestations and the combination of clinical symptoms are very heterogeneous from patient to patient, even within the same ethnic group. Some patients present with only mucocutaneous symptoms, while some suffer from systemic involvement causing serious complications.

As there are no pathognomonic clinical findings, various diagnostic criteria and classifications have been proposed during the years. According to criteria of International Study Group which was proposed in 1990, the presence of oral aphthous ulcerations and two of the following clinical manifestations is required for the diagnosis of BD: recurrent genital ulcerations, skin lesions such as erythema Nodosum-Like lesions, papulopustular lesions, ocular involvement, and positive pathergy test [9] (Table 1).

The most common presenting symptoms of the disease are mucocutaneous features [10–12]. Recurrent oral aphthous ulcerations and genital ulcerations are the most common.

Other skin lesions, such as erythema Nodosum-Like lesions, papulopustular lesions, superficial thrombophlebitis, pathergy reaction, pyoderma gangrenosum-like lesions, Sweet's syndrome-like lesions, and erythema multiforme can be observed in about 80% of the patients with BD [2, 10, 11, 13]. In addition, extragenital ulcers, palpable purpura, hemorrhagic bullae, furuncles, abscesses, perniolike lesions, and subungual infarctions, can also be seen less commonly in BD [2, 11].

Prognosis depends on the clinical involvement and the disease may result in considerable morbidity and mortality. Loss of visual acuity and neurological disease are major causes of morbidity and disability. Involvement of nervous, gastrointestinal, and large vascular systems may be lethal or can leave irreversible deficits.

Severe clinical course and systemic involvement are observed when early onset of the disease is present [2, 11, 14]. Male sex and HLA B_51_ positivity are also associated with more severe disease [2, 10, 11].

Disease course usually gets better with the passage of time with decrease in mortality rate. 

## 2. Mucocutaneous Manifestations of Behçet's Disease

### 2.1. Recurrent Oral Aphthous Ulcers

Recurrent oral aphthous ulcers (ROAUs) are a sine qua non future of BD according to the International Study Group criteria [9].

Oral aphthous ulcers frequently the first manifestation of BD recurring at least 3 times a year. It characteristically precede by many years the onset of other manifestations. They may be single or multiple and can occur after local trauma and dental intervention. The ulcer covered with grayish-white pseudo membrane or central necrotic base with round and sharp erythematous border is termed as punched-out ulcer. The ulcers are usually so painful that the patient is unable to eat during the attack. However, some patients with BD may paradoxically report no painful symptoms during active disease, despite the existence of extensive oral ulceration. The most commonly involved sites of ulcers are gingival, buccal and labial mucosa, and tongue although they can also appear in the soft and hard palate, oropharynx, and tonsils [12, 15, 16].

Minor ulcers (<1 cm in diameter) are the most (80%–85%) common form of ROAU. They are shallow, small ulcers, 1–5 in numbers, moderately painful, and heal without scarring in 4–14 days [10, 17]. Major ulcers, less common form, may be more painful and heal with scarring in 2–6 week. Herpetiform ulcers, the least common form, are numerous small (2-3 mm) and painful ulcers which may become coalescent. Uncommonly, patients may present with a mixed pattern. 

ROAU of BD should be differentiated from those of recurrent oral ulcers due to other causes.

The common causes of oral ulcer are trauma, recurrent aphthous stomatitis (RAS), infections (herpes simplex, syphilis, HIV, herpangina, primer herpetic gingivostomatitis, and hand-foot-mouth disease), mucocutaneous disease (lichen planus, erythema multiforme), immunobullous disease (pemphigus), squamous cell carcinoma, cyclic neutropenia, drugs, and systemic disorders [12, 16, 18]. Systemic conditions presenting with “aphthous-like” lesions, including BD, have been shown in Table 2: oral ulcers may be a part of systemic lupus erythematosus, MAGIC syndrome, Reiter's syndrome, and Sweet's syndrome, or may be secondary to hematinic/nutritional deficiencies (iron, vitamin and B12, folic acid, Coeliac disease) and heamatological diseases (cyclic neutropenia, lymphoma) [12, 15]. The ulcers of oral mucosa can be seen in inflammatory bowel disease, especially in Crohn's disease and, to a lesser extent, in ulcerative colitis.

In addition to a complete anamnesis and detailed review of systems, some laboratory tests such as Tzanck smear, polymerase-chain-reaction-based (PCR) assays, complete blod count, determination of serum B12, folate, and iron levels, and sometimes histopathologic examination may help diagnosis in cases with such lesions. 

In some situation, the clinical presentation of the oral ulcers of some diseases is different from that of ROAU of BD, like those in systemic lupus erythematosus: oral ulcers seen in this disease have irregular and slitlike appearance. The lesions tend to occur on the palate and often heal with a scar. Similarly, mucocutaneous lesions of Reiter's syndrome may include red patches or superficial painless mucosal erosions. The ulcers in the inflammatory bowel diseases are more likely to manifest as other types of oral ulceration. In Crohn's disease, the oral lesion are deep fissures, and they appear as linear ulcers. 

However oral ulcers, which due to other causes, are usually indistinguishable clinically from the ROAU of BD like those in RAS. RAS is a common oral mucosal condition, characterized by recurrent bouts of one or several shallow, rounded, or ovoid, painful ulcers, that recur at intervals of a few days or up to 2-3 months [16]. The minor aphthous lesions of RAS, which are most common form, often occur in the anterior part of the mouth though they can be seen at any site of oral mucosa. Although ROAU of BD cannot be distinguished clinically from common ulcers of RAS, clinical observation suggest that patients with BD tend to have more ulcers with more frequent crops, more painful and wider, and longer duration than those with RAS [18]. The patients with BD are more frequently presented with major aphthae than the patients with RAS [19]. ROAUs in BD appear to be associated with increased tissue oedema and to have an intensely erythematous border. In addition, involvement of the soft palate and oropharynx is more common in BD than in RAS [10, 17, 18].

Most patients with RAS will not develop associated disease. However, because ROAUs of BD are the most common presenting manifestation and may precede the diagnosis of BD by 6-7 years as the only manifestation, the differential diagnosis of BD must be considered in the case of any new clinical problem appear in patients with RAS. Similarly, patients with complex aphthosis also must be monitored at intervals using clinical criteria for evolution into BD. Complex aphthosis is the term used to describe the patients who have constant presence of multiple oral ulcers, or recurrent oral and genital ulcers, without systemic manifestations of BD.

Histopathologic examination of oral ulcers in BD has a nonspecific pathology with a variable infiltrate of lymphocytes, macrophages, and neutrophils at the base of the ulcer. Leukocytoclastic and lymphocytic vasculitis may be seen if the inflammation is severe [20].

Reimer et al. showed that there are no difference between direct IF on oral aphthae in RAS compared to BD [21]. They found that, compared to nonaphthous oral lesions, oral aphthae of BD and RAS are characterized by C3 deposition in the vessel walls. IgM deposits also were detected in vessel walls in some patients in both groups. 

### 2.2. Genital Ulcers

Genital ulcers (GU) are the second most commonly observed initial manifestation in BD which occur in about 80–90% of patients. They resemble oral aphthous ulcerations but are larger and deeper and have more irregular border [12, 13, 22, 23]. GU frequently heal by scarring.

They occur usually on the scrotum in males and on the vulva in females, which are most common in labia. In males, the shaft and glans penis may also be affected. Perineal, perianal, and groin lesions can occur in both sexes [10].

In females, vaginal and cervical lesions may be seen and may be associated with vaginal discharge. GU tend to be larger and deeper in female patients and sometimes lead to perforations. Large GU, frequently leave a scar, whereas small ulcers, especially those on the minor labia may heal without leaving a mark [2, 10, 11, 24]. Because ulcers are occasionally asymptomatic, patient assessment should include examination of genitalia for ulcers and scarring, even when symptoms are absent. 

Histopathologic feature of GU of BD similar to that of ROAU.

Genital ulcerations should be differentiated from venereal diseases such as syphilis, chancroid, and herpes simplex virus infection. Fix drug eruption, erythema multiforme, erosive lichen planus, otoimmun bullous dermatoses must be considered in the differential diagnosis. Recurrent genital ulcerations may also be seen in Munchausen syndrome, hypereosinophilic syndrome, myelodysplastic syndrome, tuberculosis cutis, and acquired immune deficiency syndrome [3, 4, 11]. 

### 2.3. Papulopustular Lesions

Papulopustular lesions or acnelike lesions may appear at any location and they are morphologically similar to adolescent acne. They are (28–96%) the most common cutaneous manifestation, and their distribution is more widespread than adolescent acne, affecting face, limbs, trunk, and buttocks [15, 22]. Diri et al. showed that papulopustular lesions are seen more frequently in patients with BD with arthritis [25].

The papulopustular lesions of Behçet's disease are located more often on the lower part of the body, while the lesions of adolescent acne are seen more frequently on the upper part. In addition, the pathogenesis of papulopustular lesions of Behçet's disease is different from those of acne vulgaris. According to some authors, papulopustular lesions of BD are a vasculitis, while acne vulgaris is a sebaceous gland disorder under hormonal factors. On the other hand, a controversy exists as to the histopathologic features of papulopustular lesions [12, 26–29]. These lesions are generally nonspecific also histopathologically as well as being clinically according to some authors. They advised that follicular lesions, showing suppurative folliculitis or perifollicular infiltration should be excluded, and only lesions with vessel-based and neutrophilic histologic findings should be considered as papulopustular lesions of BD [27].

Alpsoy et al. pointed out that the detection of nonfollicular lesions over the trunk or extremities, with the support of histopathologic and/or immunofluorescence studies, increases the specificity of these lesions [26]. Papulopustular lesions were included in the International Study Group Criteria as a result of their 70% sensitivity and 76% specificity; whereas some authors stated that papulopustular lesions exhibiting vessel-based neutrophilic reaction and follicle-based lesions are both features of Behçet's disease, and any papulopustular lesions, including follicular acneiform lesions, should be regarded as features of Behçet's disease [12, 28]. Boyvat considering this argument, pointed out that papulopustular lesions of BD which have nonspecific features may create problems in the diagnosis of Behçet's disease because they are extremely common also in the general population [12].

### 2.4. Erythema Nodosum-Like Lesions

The prevalence of erythema Nodosum-Like lesions was reported as 15–78% with a higher frequency in females [2, 11, 16, 30]. The lesions are tender erythematous nodules predominantly affect the lower limbs, although they can also appear at other sites, including the upper extremities, buttocks, and less commonly on the face and neck [2, 11, 15]. Often, they have more erythema and edema around the lesions than the classic erythema nodosum [31]. The lesions do not ulcer. They heal within a few weeks and usually leave a hyperpigmentation after healing [10, 15]. Recurrence is common.

Histopathological findings of erythema Nodosum-Like lesions have been reported as leukocytoclastic vasculitis, neutrophilic vascular reaction, lymphocytic vasculitis, lymphohistiocytic septal/lobular panniculitis, granulomatous panniculitis, or acute necrotizing panniculitis [30, 32, 33]. In a study, evaluating histopathological features of the nodular lesions of BD and erythema nodosum associated with other systemic diseases, it has been found that septal panniculitis, lymphocyte-predominating infiltrate in the subcutis, absence of vasculitis, and necrosis were in favor of erythema nodosum, while neutrophil-predominated infiltrate in the subcutis was more commen in BD [34]. Some authors have emphasized that early lesions show a leukocytoclastic vasculitis or a neutrophilic vascular reaction, whereas older lesions have a lymphocytic vasculitis [10, 15, 26, 27]. 

### 2.5. Superficial Thrombophlebitis

 Superficial phlebitis, which is one of the characteristics of BD, appears as painful subcutaneous nodule or stringlike hardening with reddening of the overlying skin, predominantly located on the lower extremities [2, 35]. It is segmental and can present in a characteristic migratory pattern. Although it is transient, which disappears in a few days, it has a tendency to recure. Superficial thrombophlebitis has been reported to be present in 2.2–20% of Behçet patients with higher prevalence in males [2, 10, 11]. Vena saphena magna is the most affected vein.

It can be differentiated from erythema Nodosum-Like lesions, which may be similar clinically, by dermal ultrasonography [36]: erythema Nodosum-Like lesions are hyperechoic on sonography, while the lesions of superficial thrombophlebitis are hypoechoic. 

It is important that, because of the relationship between superficial thrombophlebitis and deep venous thrombosis, close monitoring is required for the vascular systemic disease.

On histopathologic examination, organized thrombus is observed in the vein lumen. Fibrous thickening of the vein wall and sometimes infiltration of mononuclear cells may be seen [12].

### 2.6. Pathergy Test

Pathergy is the term used to describe hyperreactivity of the skin that occurs in response to any intracutaneous injection or needle prick, characterized by the formation of a sterile pustule or erythematous small papule after 24–48 hours [11]. In addition to skin pathergy test, some authors described oral pathergy test [37, 38]. The test is more strongly positive in male patients than in female [2, 39, 40]. 

Pathergy test is usually positive at the active phase of BD, though positivity is not associated with disease severity and the age of onset of BD [41]. Positivity of the test varies with geographical location: in the Mediterranean and Middle/Far Eastern countries, there is a high-pathergy positivity (40–98%) [2, 10, 11, 30]. While a positive pathergy test is an important parameter in the diagnosis of BD in these countries, the diagnostic value of the test is limited by its low sensitivity in Western countries [42, 43]. 

There are controversies about the histopathology of the pathergy reaction. Some authors found mixed infiltration, while others reported neutrophilic infiltration with leukocytoclastic vasculitis [40, 44].

### 2.7. Extracutaneous Ulcers

Extracutaneous ulcers are uncommon (3%). They are 20 to 30 mm in diameter with a yellowish necrotic base. The ulcers are recurrent and occur mainly on the internal part of the thighs, in the inguinal and axillary regions, but can affect neck, inframammary and perianal areas, breast, legs, and interdigital area of the feet [2, 10, 12, 15]. Extragenital ulcers look like aphthous ulcers and commonly heal leaving a round atrophic scar. They are common in children with Behçet's disease [10, 45]. In some cases, vasculitis had been described [45].

### 2.8. Sweet's Syndrome-Like Lesions

Sweet's syndrome-like lesions are rarely seen in patients with Behçet disease and, if present, are usually fewer in number [46]. They are seen as painful erythematous nodules and plaques, associated with fever and leucocytosis. Sometimes, they may be pustular. Sweet Syndromelike lesions can be seen on the face, neck, and extremities. 

The lesions demonstrate neutrophilic infiltration, or perivascular and periadnexal inflammatory infiltrate of lymphocytes, histiocytes, and neutrophils in the dermis. In some cases, vasculitis may also be seen [30, 46–48].

## 3. Systemic Involvement in Behçet Disease

### 3.1. Ocular Involvement

Ocular involvement in BD is frequent (28.9–80%) and is an important cause of morbidity [49]. The highest prevalence rate of the disease has been reported from Turkey and Japan [3, 4, 14]. The disease is more frequent in males than females, and males tend to have a worse visual prognosis [2, 11]. The mean age at onset of uveitis is between 20 and 30 years in male and 30 years in female patients [50].

Ocular manifestations are usually bilateral and typically occur 2 or 3 years after the onset of the disease. It may be the presenting manifestation of the disease in 10–20% of cases [4, 50]. 

It was shown that the delay between the first manifestation and eye involvement may be as long as 14years [51]. Ocular involvement carries a poor visual prognosis despite therapeutic intervention [4, 50]. The estimated risk of blindness at 5 years ranges from 15 to 25% [50].

The characteristic ocular feature is relapsing uveitis as anterior, posterior, or panuveitis, and retinal vasculitis. Anterior uveitis is frequently observed in females, whereas panuveitis is commonly encountered in males [4, 52].

Anterior uveitis with hypopyon, which has the picture of inflammatory white exudate forming a visible layer of cells in the anterior chamber is a characteristic sign of ocular BD. Hypopyon is observed only in about one-third of patients because it is transient [15, 42]. It usually disappears before the patient is seen by the physician.

The typical ocular involvement has a course with attack and remission. A single attack usually cures spontaneously without producing any sequela. When attacks become successive, they may produce sequela. The remission usually occurs so slowly that before the lesion improves, a new attack recurs which leads to severe sequelae such as synechia, cataract, and less frequently glaucoma. Successive attacks can also produce iris atrophy, atrophic retina, optic atrophy, macular degeneration, retinal veins occlusion, optic neuritis, phytisis bulbi, and loss of vision or blindness [4, 50, 53–55]. Other ocular manifestations in BD include iridocyclitis, keratitis, scleritis, episcleritis, vitritis, vitreous haemorrhage, retinal neovascularization, optic neuritis, and chorioretinal scars. However, conjunctivitis are not considered a usual feature of BD. Posterior uveitis and retinal vasculitis are the main causes for the loss of vision.

Clinical symptoms and signs include hyperemia, blurred vision, photophobia, lacrimation, floaters, periorbital, or global pain [4].

Intraocular inflammation associated with BD should be differentiated from other infectious or noninfectious causes. Uveitis may occur as a result of many conditions. A variety of infectious diseases including toxoplasmosis, herpesviruses, syphilis, tuberculosis, Lyme disease, cat scratch disease, and Whipple's disease must be ruled out by appropriate testing [4]. Development of PCR-based assays and safer methods for sampling of ocular fluids have increased the ability to diagnose infectious causes of uveitis. One of the most difficult differential diagnosis of BD is viral retinitis with anterior segment involvement. The intraocular fluids should be subjected to culture, PCR, and immunohistochemical tests for the detection of a possible viral etiology. Serologic tests for syphilis and a chest radiograph which is a useful screen for tuberculosis (and also for sarcoidosis) may be useful.

Uveitis may occur in the context of a variety of inflammatory diseases, including inflammatory bowel disease, Vogt-Koyanagi-Harada syndrome, and multiple sclerosis. Other causes of uveitis are intraocular tumors, in particular, intraocular lymphoma and reactions to medications such as cidofovir and rifabutin. Anterior uveitis and iridocyclitis in BD should also be differentiated from idiopathic uveitis, ankylosing spondylitis, Reiter syndrome, tubulointerstitial nephritis, Kawasaki disease, and sarcoidosis [4, 54].

The differential diagnosis further includes specific ocular inflammatory conditions, including Fuchs heterochromic iridocyclitis characterized by unilateral anterior uveitis with diagnostic corneal and iris changes; the “white dot syndromes” which are characterized by round white lesions involving choroid and/or retina and pars planitis characterized by a “snow bank” of inflammatory debris on the inferior pars plana. 

### 3.2. Neurological Involvement

Neurological involvement occurs in 5–10% of patients in BD. It is an important manifestation of BD because of its severe morbidity and increased mortality. It usually appears within 5 years after the onset of the disease and is more frequent in men. Central nervous system is more frequently involved than the peripheral nervous system [56, 57]. There are two types of neurological involvement: parenchymal and Nonparenchymal.

Parenchymal brain disease is more common (approximately 80%) in BD, which mainly affects the brainstem and/or basal ganglia but spinal cord lesions and hemisphere lesions may also occur. The classic manifestation is a meningoencephalitis.

All forms of neurologic manifestations have been reported in the patients with BD including headache, seizures, brainstem syndromes, cerebellar syndromes, diencephalic dysfunction, benign intracranial hypertension, ataxia, aphasia, pseudobulbar palsy, cranial nerve palsies, hemiplegia, myelopathy, and mononeuritis multiplex. Cerebellar and sensory symptoms and signs, sphincter disturbances, and behavioural changes may also be observed. Among them, pyramidal tract signs are the most frequently observed manifestations [58–60].

Some of the symptoms such as stroke, epilepsy, brain tumor, movement disorder, acute meningeal syndrome, and optic neuropathy may also be seen less commonly.

Nonparenchymal disease includes dural sinus thrombosis, arterial vasculitis, and aseptic meningitis [56]. Venous sinus thrombosis is the most frequent vascular manifestation in Nonparenchymal disease followed by cortical cerebral veins thrombosis. Other vascular manifestations include intracranial and extracranial aneurysm. In most cases, veins are much more likely to be affected than arteries. 

The most common neurologic symptom among patients with Nonparenchymal disease is headache which is caused by intracranial hypertension due to dural sinus thrombosis. Cerebral venous thrombosis may result in stroke. Stroke-like symptoms such as confusion, weakness, and dizziness may also occur.

Dural sinus thrombosis has a relatively benign prognosis in comparison to parenchymal involvement [3, 58, 59]. Involvement of parenchyma and a high protein or cell count in cerebrospinal fluid examination imply a worse prognosis in BD [15].

The differential diagnosis of neurological involvement of BD may include many diseases of the central nervous system. One of them is multiple sclerosis. Although MRI findings are distinctive in typical neurological involvement, when the predominant lesion is in the periventricular white matter, it is difficult to discriminate from the lesions of multiple sclerosis.

Other than multiple sclerosis, central nervous system infection (especially when there are cerebrospinal fluid pleocytosis and fever), cerebrovascular disease, brain tumours, and compressive myelopathy should be considered in the differential diagnosis of neuro-Beh*ç*et's disease.

Neurologic involvement of BD disease must be considered in the differential diagnosis of stroke in young adults, movement disorders, intracranial sinovenous occlusive diseases and intracranial hypertension, and other neurologic syndromes. Imaging studies and/or cerebrospinal fluid analysis may be helpful in some cases [61–63]. MRI differentiates parenchymal lesions from the Nonparenchymal forms [63]. Diffusion-weighted imaging may be useful in the case of stroke-like episodes, revealing an increase in the diffusion coefficient in BD lesions. The clinical similarity between successive attacks may also be helpful for diagnosing BD. Cerebrospinal fluid glucose determination may be a useful parameter to differentiate central nervous system involvement in BD from other diseases, particularly infections [63, 64].

Histopathological studies, investigating central nervous system involvement, have mainly demonstrated that there is a perivascular lymphocytic infiltration with areas of necrosis in BD [64–67]. It is uncertain whether the areas of necrosis are caused by vasculitis or inflammatory infiltrate around the small vessels.

Riera-Mestre et al. have found perivascular lymphocytic infiltration with reactive astrocytosis, but no frank vasculitis in the brain biopsy specimens in their study [64]. They suggest that the absence of endothelial degeneration supports a perivascular inflammatory process rather than frank vasculitis.

### 3.3. Gastrointestinal Involvement

The frequency of gastrointestinal system involvement is variable in different countries [4, 15]. In Japan and Korea, the prevalence of gastrointestinal system involvement is higher (15–45%) than that in the Middle East and Mediterranean [15, 68, 69]: gastrointestinal manifestations occur in one-third of Japanese patients, while in Turkey and Israel, the prevalence is about 0–5% [2, 49]. There is no significant difference in the frequency of gastrointestinal involvement between male and female [2, 11].

Gastrointestinal system manifestations can occur throughout the gastrointestinal tract, from the esophagus to the anus. The ulcers are most commonly found in the terminal ileum, followed by the caecum and other parts of the colon [70]. The ileocaecal ulcers have a distinct tendency to perforate. They may lead to symptoms of abdominal pain, diarrhea, or constipation and proctorrhagia as well as the acute abdomen, which can be caused by perforation of ulcers [31, 71].

Gastritis, peptic ulcers, abdominal pain, dyspepsia, vomiting, and diarrhea may be due to stomach and small intestine ulcers, while dysphagia, retrosternal pain, and hematemesis are due to esophageal ulcers.

The differential diagnosis of BD from inflammatory bowel disease is sometimes difficult and challenging. The main differential diagnosis of lower intestinal lesions is with Crohn's disease. Both diseases have the same ulceration. It was shown that ulcers in BD were usually round or oval, while in Crohn's disease, they were essentially longitudinal. If there is a longitudinal ulcer in BD, the disease has focal distribution, otherwise if distribution is segmental or diffuse, it is Crohn's disease [31, 72]. In addition to ulcers, granuloma formation in intestinal lesions suggests Crohn's disease, which is not seen in BD [73]. The association of RAS with coeliac disease is well established. It has been suggested that up to 5% of patients with RAS have gluten-sensitive enteropathy [16, 74].

### 3.4. Articular Involvement

Articular involvement is seen in approximately 30–70% of BD and may be the first manifestation of the disease in about 16.5% of them [2]. It affects commonly knees, ankles, wrists, and elbows [2, 11]. Articular involvement is observed in the form of arthralgia, arthritis, and synovitis. Arthralgia is mainly of inflammatory type. Joint disease, which can be symmetrical, usually mono- or oligoarticular and heals in few weeks, but may take several weeks or months to heal. It is usually transient, nonerosive, and nondeforming. Chronic or polyarticular arthritis and osteonecrosis can be seen occasionally. Ankylosing spondylitis is not seen in BD for some authors, while for others it is related to BD [3, 31]. Sacroiliitis and involvement of the spine are not among the common manifestations of BD, which can serve as differential diagnosis for Reiter's syndrome.

Articular involvement may be confused with seronegative arthropathies, rheumatoid arthritis, and psoriatic arthritis. When the joint manifestations are acute and transient, the disease resembles to rheumatic fever. Chronic and polyarticular form, which is exceptional, mimics rheumatoid arthritis. However, the articular changes of rheumatoid arthritis are destructive in character [75]. Synovial fluid analysis and synovial biopsies may help diagnosis by determining the cell type which may differentiate arthritis of BD from the rheumatoid arthritis. Serological abnormalities are seen in inflammatory arthropathies. Seronegative arthropathies have mainly psoriasiform skin lesions, aortic insufficiency, frequent axial involvement and peripheral enthesopathies, and sacroiliitis. 

### 3.5. Vascular

BD is a systemic vasculitis affecting virtually all types and sizes of vessels. The prevalence of vascular involvement is about 1.8–33% and was found higher in male patients than females [2, 4, 31].

Venous system involvement is more common than arterial system involvement. Venous involvement results in both superficial thrombophlebitis and deep venous thrombosis. Venous thrombosis affects extremities, mainly lower limbs. Large vein thrombosis is less frequent than deep vein thrombosis of limbs. It involves mainly superior and inferior vena cava, but may also affect mesenteric, portal, hepatic, splenic, renal, dural sinus, jugular, subclavian axillary, and iliac veins. Occlusion of suprahepatic veins, which is rare, causes a Budd-Chiari syndrome, leading to mortality [76, 77]. In addition to Budd-Chiari syndrome, thromboses of the superior and inferior vena cava and of dural sinuses are associated with a poor prognosis [15, 77, 78]. Hughes-Stovin syndrome should be differentiated from BD in which deep venous thrombosis, often involving the vena cava, is seen accompanied by single or multiple pulmonary arterial aneurysms. 

Arterial lesions are less frequently observed in Behçet patients. Arterial involvement includes thrombosis, aneurysms, and pulse weakness and affects pulmonary, iliac, popliteal, femoral, and carotid arteries. Pulmonary arterial aneurysms, which occur in 1% of patients, have a high mortality rate. The main symptom is haemoptysis, and these patients usually have associated thrombophlebitis and deep venous thrombosis [3]. 

The arterial involvement in BD resembles those of Takayasu's arteritis, including aneurysm formation and arterial occlusion. It is suggested that arterial involvement of BD may result from neutrophilic vasculitis, which targets the vasa vasorum [79]. Accordingly, on histopathological studies, it was demonstrated that the number of vasa vasorum infiltrated with neutrophils and lymphocytes was significantly increased in vasculo-Behçet's disease compared with that in Takayasu's arteritis and other inflammatory aneurysms [79].

### 3.6. Pulmonary Involvement

Pulmonary involvement is rare in BD (0.7–7%) [2]. It has a higher frequency in males than females [2, 11].

Pulmonary manifestations have different etiology: vasculitis, embolism, fibrosis, pleurisy, and infection. The manifestations are mainly related to vasculitis of the pulmonary arteries, veins, and septal capillaries. Pulmonary vascular involvement can lead to aneurysm formation (aortic or pulmonary artery aneurysm), thrombotic occlusion, (mainly in the vena cava), haemorrhage, pleural effusion, pulmonary infarct, and focal or diffuse pulmonary fibrosis. Aneurysms are more common than thrombosis and tend to be multiple [31]. Although venous system involvement is more common than arterial system involvement, rupture of an arterial aneurysm is a significant cause of mortality in BD. The most common and predominant symptom of pulmonary arterial aneurysm is haemoptysis. 

It must be known that detection of a pulmonary aneurysm in the setting of a vasculitic illness is highly suggestive of BD, which is found rarely in other forms of vasculitis [17, 80]. 

### 3.7. Cardiac Involvement

Cardiac involvement is uncommon in BD. Multiple case reports can be found in the literature on cardiac manifestations, describing every form of cardiac involvement such as myocarditis, valvular lesions, pericarditis, ventricular aneurysms, intracardiac thrombosis, coronary vasculitis, and many others [15, 81]. Currently, it was shown that atherosclerosis is probably not increased in Behçet's disease, unlike rheumatoid arthritis and systemic lupus erythematosus [2, 3, 82].

### 3.8. Genitourinary System Involvement

Renal involvement is not frequent in BD and is usually transient. Sometimes they may become chronic. Hematuria, proteinuria, leukocyturia, and rarely cast may be seen in the patients with renal involvement of BD. Urethritis is not a feature of BD that may facilitate to distinguish it from Reiter's syndrome [17, 31].

Orchitis and epididymitis can also occur in patients with BD [31]. They have a low tendency for recurrence. The attack of epididymitis may be a painful or a painless swelling, but the attack of orchitis, which affects both testicles, is painful. Attacks last for few days or weeks. 

## Figures and Tables

**Table 1:** International Study Group criteria for the diagnosis of Behçet's disease [9].

Recurrent oral ulceration	Minor/major aphthous or herpetiform ulcer observed by the physician or patient which recurred at least three times in one 12-month period
Plus two of the following:	
Recurrent genital ulceration	Aphthous ulcer or scarring observed by the physician or patient
Eye lesions	Anterior/posterior uveitis, cells in the vitreous on slit-lamp examination or retinal vasculitis observed by an ophthalmologist
Cutaneous lesions	Erythema nodosum observed by physician or patient, pseudofolliculitis or papulopustular lesions, or acneiform nodules observed by physician in postadolescent patients not receiving corticosteroids
Positive pathergy test	Interpreted by the physician at 24–48 h

**Table 2:** Systemic conditions presenting with aphthous-like lesions.

Behçet's disease
Gastrointestinal disorders
Nutritional/Heamatological deficiencies
Heamatological diseases
MAGIC syndrome
Reiter syndrome
PFAPA syndrome
Sarkoidoz
Drug reactions
